# Striated Muscle Regulation of Isometric Tension by Multiple Equilibria

**DOI:** 10.1371/journal.pone.0008052

**Published:** 2009-12-08

**Authors:** Henry G. Zot, Javier E. Hasbun, Nguyen Van Minh

**Affiliations:** 1 Department of Biology, University of West Georgia, Carrollton, Georgia, United States of America; 2 Department of Physics, University of West Georgia, Carrollton, Georgia, United States of America; 3 Department of Mathematics, University of West Georgia, Carrollton, Georgia, United States of America; George Mason University, United States of America

## Abstract

Cooperative activation of striated muscle by calcium is based on the movement of tropomyosin described by the steric blocking theory of muscle contraction. Presently, the Hill model stands alone in reproducing both myosin binding data and a sigmoidal-shaped curve characteristic of calcium activation (Hill TL (1983) Two elementary models for the regulation of skeletal muscle contraction by calcium. Biophys J 44: 383–396.). However, the free myosin is assumed to be fixed by the muscle lattice and the cooperative mechanism is based on calcium-dependent interactions between nearest neighbor tropomyosin subunits, which has yet to be validated. As a result, no comprehensive model has been shown capable of fitting actual tension data from striated muscle. We show how variable free myosin is a selective advantage for activating the muscle and describe a mechanism by which a conformational change in tropomyosin propagates free myosin given constant total myosin. This mechanism requires actin, tropomyosin, and filamentous myosin but is independent of troponin. Hence, it will work equally well with striated, smooth and non-muscle contractile systems. Results of simulations with and without data are consistent with a strand of tropomyosin composed of ∼20 subunits being moved by the concerted action of 3–5 myosin heads, which compares favorably with the predicted length of tropomyosin in the overlap region of thick and thin filaments. We demonstrate that our model fits both equilibrium myosin binding data and steady-state calcium-dependent tension data and show how both the steepness of the response and the sensitivity to calcium can be regulated by the actin-troponin interaction. The model simulates non-cooperative calcium binding both in the presence and absence of strong binding myosin as has been observed. Thus, a comprehensive model based on three well-described interactions with actin, namely, actin-troponin, actin-tropomyosin, and actin-myosin can explain the cooperative calcium activation of striated muscle.

## Introduction

For vertebrate striated muscle, modeling steady-state isometric tension data with the known properties of calcium binding has proven difficult to achieve. The tension response to varying calcium is distinctly sigmoidal, suggesting an underlying cooperative mechanism. A potential basis for cooperative activation is the association of myosin with thin filaments [1], [2]. All present models of striated muscle regulation were derived originally from fitting myosin binding to thin filaments at fixed calcium [2]–[9]. An allosteric mechanism based on seven myosin binding sites has been proposed [1], [10], but a strictly allosteric model must be reconciled with the muscle lattice, which allows only 1–2 myosin bound per structural repeat [11], [12]. In addition, calcium rather than myosin varies in the muscle. Given these restrictions, cooperative calcium binding has been proposed as a mechanism for activating muscle contraction [8], [9]. However, direct measurements of calcium binding have been consistently documented to be non-cooperative both in the presence and the absence of myosin [13], [14].

Thin filaments consist of continuous parallel strands of polymeric actin and tropomyosin molecules (c.f. [15] for review). Two strands of the actin polymer bind side-by-side along their length to form a single double-stranded helical structure, and one strand of tropomyosin (Tm) is located along each side of the actin helix. In striated muscle, one calcium-binding troponin molecule (Tn) is bound to each subunit of Tm. A linear group of seven actin monomers, one Tm subunit, and one Tn, corresponding in length to the pitch of the actin helix constitute a structural repeat. There are 26 structural repeats, each defined by the length of a Tm subunit, in series along the length of a thin filament (1000 nm). Approximately 20 Tm subunits (75% of thin filament) are overlapped by thick filaments in the muscle lattice at rest.

Thin filament activation, which gives rise to isometric tension, is defined as the exposure of myosin binding sites [16] by the movement of Tm away from a position that blocks these sites on the actin filament. Owing to an extended structure, each subunit of Tm regulates the interaction of up to seven potential myosin molecules with binding sites of actin (one site per actin monomer). The flexibility of the Tm polymer allows each subunit to occupy any of three discreet positions relative to the location of the myosin binding site on the outer face of the actin helix. Positions B, C, and M of Tm correspond to blocking, central, and myosin-dependent respectively [17]. Tm in Position B completely blocks myosin binding and is favored at low calcium [18]. Increasing calcium shifts the distribution of Tm away from Position B to favoring Position C [19]. Structural reconstructions of Tm in Position C reveal a partial overlap with the myosin binding site [20], but Tm is expected to undergo extensive thermal motions about Position C, which would expose the myosin binding site [21]. Myosin binding displaces Tm to Position M regardless of calcium or the starting position of Tm [22].

Each of the positions of Tm can be related to a specific biochemical interaction with actin, namely, actin-Tn, actin-Tm, and actin-Tm-myosin for Positions B, C, and M respectively. For Position B, an interaction between Tn and actin [23] can only occur when the complex of Tm and Tn (Tmn) is located in this position [24]. Calcium binding to Tn weakens the Tn-actin interaction [23], which is consistent with the change in the distribution of the Tmn complex toward Position C [19]. The displacement of Tmn to the inner domain of actin prevents the Tn-actin interaction [25]. For Position C, a direct interaction between Tm and actin is consistent with the position of Tm in Tm-decorated actin filaments (no Tn and myosin) and with a favorable orientation of Tm that promotes multiple electrostatic interactions between actin and Tm in this position [20]. For Position M, the association of myosin enhances the affinity of Tm for actin [1] and, reciprocally, Tm enhances the affinity of myosin for actin [6]. These observations are consistent with the formation of a ternary complex of myosin, Tm, and actin. That this complex may induce structural changes in Tm is suggested by reconstructions of myosin-decorated thin filaments showing a contiguous length of unsupported Tm in Position M beyond the last bound myosin [22]. A stiffening of Tm in Position M would enable free access to myosin binding sites, as has been modeled previously [3]. In Positions C and M, Tn is disengaged from actin but remains tethered to Tm. Coupling of chemical binding energy to the work associated with the position of Tm has been described for muscle regulation [3], [26].

The Hill model [26] stands alone in being capable of explaining sigmoidal calcium activation [9], [26]. Rather than strictly an allosteric mechanism, interactions between Tm subunits enhance myosin binding [3]. Both calcium and myosin binding perturb nearest neighbor Tm interactions and thereby contribute to the movement of Tm [3], [26]. To achieve cooperative activation by a nearest neighbor mechanism, calcium binding must not only promote the movement of Tm and also alter interactions between nearest neighbor Tm subunits [26]. Whereas calcium binding to Tn has been shown to alter the distribution of Tm subunits between Positions B and C [19], experimental evidence for altered interactions between Tm subunits remains to be established [27].

As a result, tension data have not been analyzed with the aid of a comprehensive quantitative model even when the results are purported to be consistent with a nearest neighbor mechanism [28]. To address the possibility that Tn does not regulate interactions between nearest neighbor Tm subunits, we examined whether it is reasonable for calcium activation to be modeled solely on the basis of well-established protein interactions of the thin filament. The model would have to provide a biochemical basis for the known positions of Tm but be consistent with the following data: only 1–2 myosin are bound per Tm subunit at maximum calcium, calcium binding is non-cooperative at fixed myosin, the binding of myosin is cooperative at fixed calcium, and activation by calcium is highly cooperative. We demonstrate a model, consistent with steric blocking theory [29], [30], that meets these requirements and fits a challenging set of isometric tension records.

## Analysis

### Description of Model

#### Mechanism of cooperative activation

**Figure 1:**
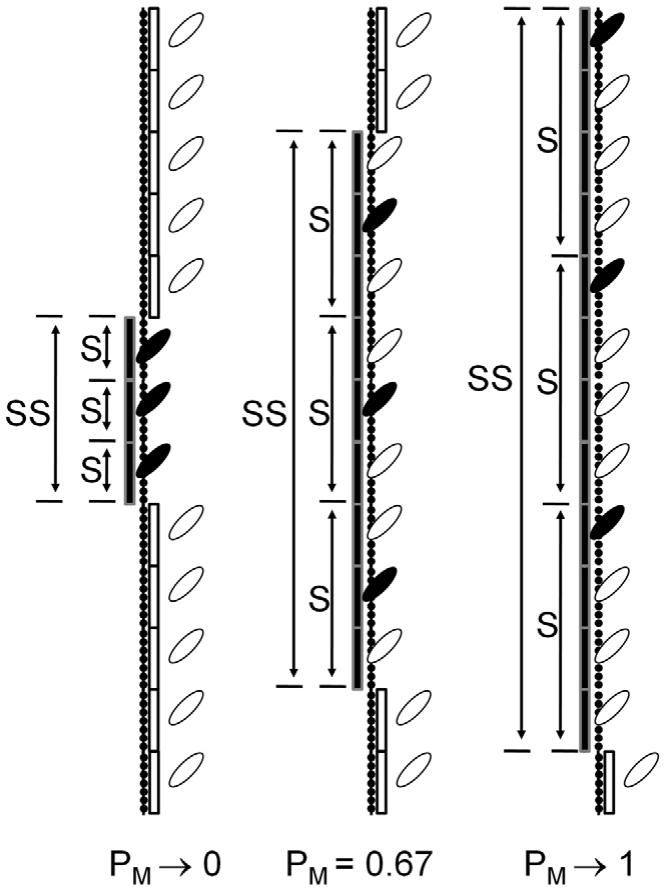
Novel myosin-based cooperative mechanism for vertebrate striated muscle. The diagram depicts positions of tropomyosin (Tm) generated by interactions of troponin (Tn) and myosin with actin. An interaction between Tn and actin (not depicted) is energetically coupled to the stability of Tm in Position B (open rectangle), which blocks the association of myosin (open myosin head) with actin (filled monomers). An interaction of myosin with actin and Tm (closed myosin head) is coupled to a conformational change in Tm and the stability of Tm in Position M (closed rectangle). The conformational change stiffens one or more Tm subunits into a functional unit, referred to as a segment (S), which requires one coupled myosin and excludes all other myosin bound within S from being coupled. The stiffening of Tm requires multiple myosin to be coupled, but, since S can have only one coupled myosin, myosin from multiple S must cooperate to form a larger functional unit of Tm, referred to as a super segment (SS). Only one bound myosin per Tm subunit has the potential to be coupled within a segment, referred to as free myosin (open head attached to actin). Free myosin stabilizes the coupled state of myosin by being available to be coupled, as coupling within the segment is dynamic. The number of Tm subunits per S depends on the probability that myosin can be coupled (P_M_). The maximum number of Tm per S and the number of myosin that must be coupled to form SS are intrinsic properties of Tm arbitrarily chosen to be 4 and 3 respectively for this diagram.

We propose a cooperative mechanism of activation based on the formation of a myosin-induced conformation of Tm and the propagation of this structure along the Tm polymer (Fig. 1). The propagation results from two seemingly contradictory properties of Tm, namely, that Tm adopts a rigid structure in Position M to couple only one myosin at a time and that stability of the rigid structure requires simultaneous coupling of multiple myosin. Contiguous Tm subunits form segments and multiple segments work together to form a super segment; neither the individual segments nor the super segment can exist without the other. Because an isolated segment can have only one coupled myosin, the multiple myosin required for super segment formation must be in separate segments.

A super segment expands as a function of the probability that coupling will occur, designated P_M_. P_M_ depends on the stability of the segments, which, in turn, depends on *free myosin* being available within the segment to accept coupling dynamically. The structure of the thick filament is precisely calibrated to deliver at least one myosin head to the domain of each Tm; on average, 1.3 myosin heads are bound for each Tm subunit at maximum isometric tension in insect flight muscle [11]. Although any of the uncoupled myosin within a segment could be free, the pool of free myosin is limited to one myosin per Tm subunit by the rigid structure of Tm. Thus, the coupling reaction and resultant conformational change in Tm fixes the total mole fraction of coupled and free myosin equal to the mole fraction of tropomyosin. Maximum expansion of a segment is determined by the ability of unsupported Tm in Position M to resist the tendency to return to equilibrium in Position C, and, hence is an intrinsic property of Tm. Similarly, the intrinsic property of Tm determines the number of coupled myosin in separate segments required to induce the rigid conformation of the super segment. The blocking of myosin by Tm in Position B regulates P_M_ in vertebrate striated muscle.

#### Description of the system

**Figure 2:**
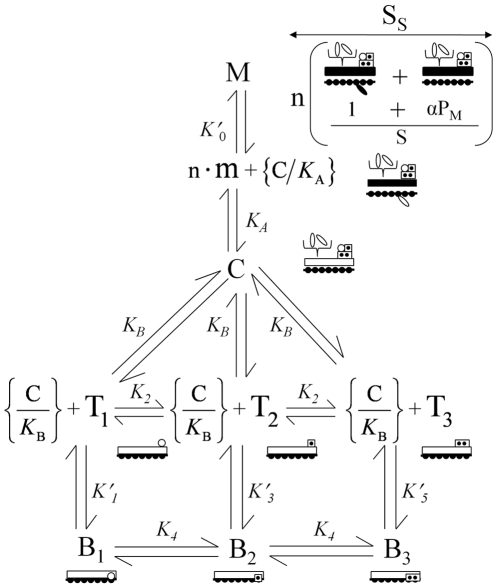
Annotated equilibrium model. B_1_, B_2_, B_3_, C, and M represent mole fractions of binding states coupled to the positions of Tm denoted by the letters (blocking, central, and myosin dependent, respectively). B_1_, B_2_, and B_3_ are represented by Tm (open rectangle) held in a blocking position on actin by interactions between actin and Tn in each of three possible calcium bound states, namely, zero sites filled (open circle), one site filled (one dot), and two sites filled (two dots). Tn held by Tm in Positions C and M is uncoupled. T_1_, T_2_, and T_3_, represent the mole fractions of uncoupled Tn with zero, one, and two calcium bound respectively (single symbol with open circle, one dot, and two dots). T_1_, T_2_, and T_3_ must be carried by thermal motions of Tm to the vicinity of actin binding sites in Position B for coupling to occur. The mole fraction of Tm in Position B, {C/*K*_B_} (brackets denote non-equilibrium state), determines the mole fraction of actin binding sites available for interaction with T_1_, T_2_, and T_3_ (circle, one dot, and two dots dissociated from actin in Position B). The segment conformation of Tm (filled rectangle) requires the formation of a coupled myosin state (closed myosin head). The coupled myosin state is stabilized by the mole fraction of free myosin present in segments (open myosin head attached to actin) and the mole fraction of C transiently present in Position M (given by {C/*K*_A_}). Each segment is composed of a variable number of Tm subunits (1+αP_M_; P_M_ is the probability of the M state) and a super segment is composed of *n* segments. The mole fractions of segments (S) and super segments (S_s_) each equal M. Tm in Positions C and M supports cycling myosin intermediates (pair of myosin heads) for sliding filaments and isometric tension respectively. Omitted from the diagram for clarity are the redundant reactions for calcium binding to Tn in Positions C and M and the explicit reaction with actin that forms free myosin.

We describe a single thermodynamic system coupled to the positions of tropomyosin. We assign the work associated with specific positions of Tm to the binding energies of biochemical interactions known to take place in these positions. The stability of the Tmn complex in Position B is assumed to be dominated by the affinity of the Tn-actin interaction. Because calcium binding to Tn destabilizes the Tn-actin interaction, a discreet coupled state exists for each of the calcium bound state of Tn. Given two regulatory sites for calcium, three actin-bound states of Tn couple Tmn to Position B, namely, ATmn.Ca_0_(B), ATmn.Ca_1_(B), or ATmn.Ca_2_(B), for Tn with zero, one or two bound calcium respectively. Interactions with actin in Positions C and M occur independently of Tn and have undefined calcium binding states (X); Position C is stabilized by an interaction between Tm and actin, ATmn.X(C), and Postion M is stabilized by a ternary complex of myosin, Tm, and actin, ATmn.X(M). The mole fraction of each state, ATmn.Ca_0_(B), ATmn.Ca_1_(B), ATmn.Ca_2_(B), ATmn.X(C), and ATmn.X(M), is represented by B_1_, B_2_, B_3_, C, and M respectively (Fig. 2). These five states constitute the system.

The system is open with respect to Tn, so that the coupled Tn bound to actin exhanges with uncoupled Tn, which is dissociated from actin. The fact that uncoupled Tn is irreversibly tethered to Tm results in the sequestration of uncoupled Tn from Position B in states C and M, which has two significant consequences. First, the sum of the mole fractions of uncoupled Tn, corresponding to 0, 1, and 2 calcium bound (T_1_, T_2_, T_3_; Fig. 2), equals the sum of C and M. The mole fractions of coupled and uncoupled Tn states can be calculated by solving the partially overlapping multiple equilibria of coupled and uncoupled Tn states. Second, transitions of Tn between Positions C and B ({C/K_B_}; Fig. 2) are independent of Tn yet control the availability of actin binding sites for interaction with T_1_, T_2_, and T_3_ in Position B. Hence, the formation of state B_1_, B_2_, or B_3_ depends on the mole fraction of available actin binding sites ({C/K_B_}) and the mole fraction of uncoupled Tn (T_1_, T_2_, T_3_), which are determined by separate reactions (Fig. 2). Thus, the reaction that couples Tmn to Position B is second order.

Similarly, exchange of myosin between coupled and free states makes the system open with respect to myosin. The exchange between coupled and free myosin takes place in segments of Tm (Fig. 1). Only one of the myosin heads bound per Tm subunit [11] can couple with a segment owing to the conformation of Tm. The mole fraction of free myosin can be calculated from the mole fraction of Tm subunits forming segments (m; Fig. 2). Segments form independently of myosin by a thermally driven process ({C/K_A_}; Fig. 2) and free myosin forms by a separate interaction with strong binding sites of actin, hence, the coupling reaction is second order. Free myosin stabilizes the coupled state but is uncoupled from the energy required to maintain Tm in Position M.

#### Summary of activation

Given that myosin binding sites of actin are fully blocked by Tm in Position B and fully exposed when Tm is in Position M [17], activation of the thin filament is functionally *off* when Tm is in Position B and functionally *on* when Tm is in Position M. The displacement between thin and thick filaments by sliding filaments is expected to destabilize the interaction between myosin and actin and, hence, Position M. Thus, isometric conditions of the muscle are required for maximal coupling of myosin and stability of the super segment. Sliding filaments may be supported by Tm in Position C because the weak interaction between Tm and actin may allow Tm to move away from a blocking position faster than the cycling rate of the myosin in isotonic conditions. Although we avoid these complications by focusing on isometric condition here, our model is sufficiently robust to allow for a future analysis of sliding filaments.

#### Limitations and constraints

The mass action relationships that we derive account for the distributions of coupled and uncoupled states of a self-consistent macroscopic thermodynamic system. Although this system is consistent with the structural model we propose, other structural interpretations may be consistent with the same bioenergetics. The derived macroscopic constants are measurable but, more work will be required to fully understand the molecular events that determine the spatial arrangement of coupled and uncoupled myosin in relation to the structure of Tm in Position M. Microscopic reversibility is assured by the derivation in the following section of explicit conservation relationships and by assuring that the conservation relationships, 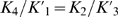 and 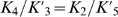 (Fig. 2), are maintained in computations using the model.

### Derivation of Model

#### Conservation of coupled states

To derive the conservation of mass relationships for the coupled states, let U_T_ represent the total number of Tm subunits in a given preparation. If the number of Tm subunits (U) per segment (S) is defined with a parameter *g*, (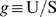), then the total mass (or number) of Tm subunits (U_T_) distributed among the coupled states is given by
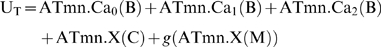
(1)where 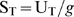 represents the total number of segments in a given preparation. Letting B_1_, B_2_, B_3_, C, and M equal 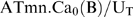, 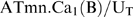, 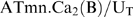, 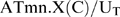, and 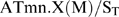 respectively (Fig. 2), expresses the model in terms of mole fractions and Eq. 1 becomes
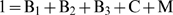
(2)From the discussion of segments above and (Model Description; Fig. 1), it can be seen that every segment has exactly one coupled myosin, hence, 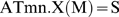 and the mole fraction of segments that have formed, 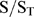, is given by M, thus, 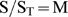.

#### Conservation of uncoupled states

Letting Tn.Ca_0_, Tn.Ca_1_, and Tn.Ca_2_, represent the mass of uncoupled Tn states with 0, 1, and 2 calcium bound respectively, the mole fractions of these states are given by 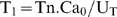, 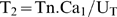, and 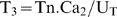 (Fig. 2). Substituting T_1_, T_2_, and T_3_ for C and M in Eq. 2 gives the relationship for the conservation of Tn,
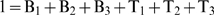
(3)Letting Y_T_ represent the total mass of strong binding myosin and 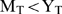, then M_T_ represents the mass of myosin capable of being coupled to Tm in Position M. If m_f_ represents the free myosin (see definition in Model Description; Fig. 1), then the relationship
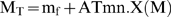
(4)accounts for all non-extraneous biochemical and structural intermediates of myosin. Given delivery of at least one myosin per Tm subunit by the thick filament [11] and the limit of one coupled myosin per Tm subunit (property of Tm described in Model Description; Fig. 1), M_T_ is a constant; 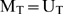. Thus, of the myosin that can be coupled, the mole fraction present in segments is given by 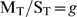 and, thus
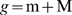
(5)where 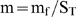 and 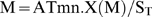 represent the mole fractions of free myosin and coupled myosin that are present in segments respectively. Although uncoupled, free myosin is bound to the thin filament (Fig. 1).

#### Equilibrium relationships for position M

The formation of the super segment depends on both the free myosin (m; Fig. 2) and the mole fraction of Tm that is available for coupling in Position M, given by 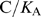 (Fig. 2). As 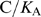 and m are independent, the reaction proceeds by mass action given by 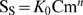 where 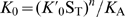, *K*′_0_ and *K*_A_ are intrinsic constants, S_S_ represents the mole fraction of Tm that has formed super segments, and the parameter, *n*, represents the number of myosin that must be coupled simultaneously in order for the super segment to form. Letting SS and SS_T_ represent a super segment and the total mass of super segments respectively, then 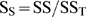. As 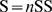, there is a proportional relationship between segments and super segments, hence 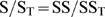. Because 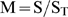 (see above), 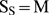 (Fig. 2), from which the following is derived
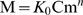
(6)By substitution for m (Eq. 5) in Eq. 6,
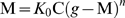
(7)From Eqs. 5–7 free myosin can be seen to vary as a function of segment size. By contrast, the only other model shown capable of generating a cooperative activation by calcium assumes that free myosin is a constant determined by the muscle lattice ([26]; also see Discussion).

To account for variable segment size, we make *g* dependent on P_M_ (see definition in Model Description above; Fig. 2) and a parameter *α* that determines the maximum segment length in subunits of Tm, i.e., 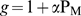. When 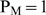, *g* is maximum, 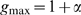, and the maximum super segment length is given by 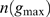.

Because M represents the mole fraction of Tm subunits that are in Position M, M is also a Bayesian probability that myosin will be coupled to the position of Tm. Thus, 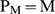, which by substitution above gives
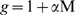
(8)Substituting Eq. 8 into Eq. 7 results in a relationship that can be evaluated given experimental parameters *n* and *α*,
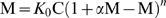
(9)We note that if 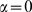, the maximum segment size is one Tm subunit and M goes to zero as a function of M, i.e., coupling is a self-limiting process. Thus, there is a selective advantage for 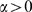. (A more detailed analysis suggests that the selective advantage is for 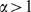.)

#### Equilibrium relationships for coupled and uncoupled Tmn

The expressions for calculating the mole fraction of coupled Tmn are derived from a two-step sequence that includes calcium-independent movement of Tm between Positions B and C governed by *K*_B_ and interaction between Tmn and actin in Position B governed by one of three possible stability constants, *K*′_1_, *K*′_3_, and *K*′_5_ (Fig. 2). These mass action equilibria can be represented by
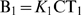
(10)

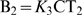
(11)

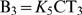
(12)where *K*_1_, *K*_3_, and *K*_5_ are all first order constants composed of the following: 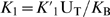, 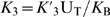, and 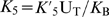 (Fig. 2).

Similarly, the expressions for calculating the mole fractions of calcium bound Tmn are derived from the pathways for calcium binding to uncoupled and coupled states (Fig. 2).
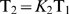
(13)

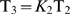
(14)

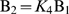
(15)and
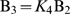
(16)where *K*_2_ and *K*_4_ are defined as 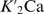 and 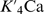, respectively. *K*_2_ and *K*_4_ allow for the input of calcium, Ca (Table 1).

**Table 1:** Summary of Dependent and Independent Variables.

Vari-able	Equivalent	Comments
B_1_	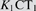	Mole fraction of Tm subunits coupled to the Tn-actin interaction and no calcium bound. Tm in Position B.
B_2_	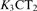 ; 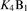	Mole fraction of Tm subunits coupled to the Tn-actin interaction and one calcium bound. Tm in Position B.
B_3_	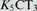 ; 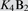	Mole fraction of Tm subunits coupled to the Tn-actin interaction and two calcium bound. Tm in Position B.
B^−^	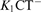	Mole fraction of Tm subunits coupled to the mutant Tn-actin complex. Tm in Position B.
T_1_	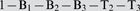 ; 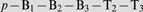	Mole fraction of Tn dissociated from actin and tethered to Tm; no calcium bound. Tm position is undefined.
T_2_	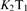	Mole fraction of Tn dissociated from actin and tethered to Tm; one calcium bound. Tm position is undefined.
T_3_	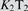	Mole fraction of Tn dissociated from actin and tethered to Tm; two calcium bound. Tm position is undefined.
T^−^	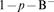	Mole fraction of mutant Tn dissociated from actin and tethered to Tm.
C	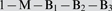	Mole fraction of Tm directly associated with actin. Tm at equilibrium in Position C.
M	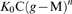	Mole fraction of Tm subunits coupled to the myosin-actin interaction. Tm in Position M.
P_M_	M	Probability of myosin coupled to the work associated with Tm in Position M; 0≤P_M_≤1
Ca		Calcium concentration; continuously independent variable.

#### Fitting mutant troponin data

We test the ability of our model to simulate the results of replacing wild-type Tn with mutant Tn unable to bind calcium [28]. To model the replacement of wild-type Tn, we introduce ATmn^−^(B) and Tn^−^ to represent coupled and tethered states that contain mutant Tn respectively. Letting B^−^ and T^−^ represent 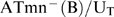 and 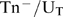, respectively, we derive the following,
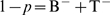
(17)where the parameter, *p*, represents the mole fraction (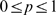) of total Tn that is wild type. Assuming that mutant Tn, which cannot bind calcium, associates with actin like wild-type apo-Tn (T_1_), the equilibrium relationship for the association of mutant Tn and actin is given by Eq. 10, hence 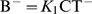. By substitution into Eq. 17, we obtain the following relationship for evaluation,
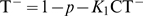
(18)We note that *K*_1_ in Eq. 18 is that of wild-type apo-Tn, although this is a simplifying assumption without experimental support.

The conservation equations for mutant and wild-type coupled states and wild-type Tn states respectively are
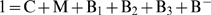
(19)

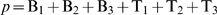
(20)


#### Calculation of calcium activation

To minimize the number of simultaneous equations to solve, we substituted equivalent expressions (Table 1) into Eqs. 19, and 20, to derive
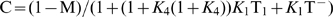
(21)and
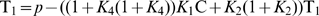
(22)The solution of Eqs. 9, 18, 21 and 22 for an arbitrary calcium concentration and mole fraction of wild type Tn yields values for variables C, M, T_1_, and T^−^. From these values, all other variables are evaluated using the relationships in Table 1.

#### Expressions to fit myosin binding data

We develop the relationships necessary to fit the cooperative binding of myosin as detected by a change in fluorescence of modified Tn in reconstitution experiments [16]. The myosin-dependent fluorescence change in the absence of calcium is represented by two sequential reactions, 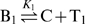 and 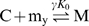, where m_y_ is the measured free myosin in solution and *K*_1_ and 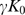 are equilibrium constants that govern each reaction respectively. Because myosin is more constrained in the muscle lattice than in solution, we use the factor, γ, to correct *K*_0_ for the solution behavior of m_y_ in the reconstitution experiments [16]. The fluorescence change is assumed to result from an increase of T_1_ by the first reaction, in response to myosin association in the second reaction. This sequence is consistent with a direct correlation between the fractional fluorescence change, ΔF, and the formation of the coupled state, M. Letting 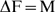 and 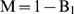, the relationship between the fluorescence change and free myosin is given by
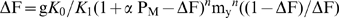
(23)where P_M_ = ΔF. A fit of the fluorescence data with Eq. 23 requires solving an *n^th^* order polynomial. This formidable task is avoided by reversing the dependent variable of the data and transforming Eq. 23 accordingly, as follows
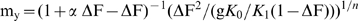
(24)Equation 24 is used to derive 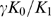 from myosin-dependent fluorescence data, given values for *n* and *α*.

#### Total myosin binding

In our model, total equilibrium binding of myosin (Θ) is the sum of cooperative (ΔF) and non-cooperative (θ) binding. Each segment of 7(*α*+1) myosin binding sites requires one coupled myosin that binds cooperatively to one myosin binding site. Once formed, a segment provides 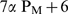 actin binding sites for non-cooperative binding. Given P_M_ = ΔF, the following relationship accounts for the total myosin bound as a function of free myosin.
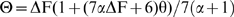
(25)where ΔF is generated by Eq. 23 and θ is generated by the following relationship for simple saturation binding.
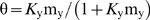
(26)where *K*_y_ is the measured association constant for strong binding myosin with pure actin.

## Results

Experimental observations and energy conservation place constraints on the equilibrium constants, *K*_0_, *K*_1_, *K*_3_, *K*_5_, *K*′_2_, and *K*′_4_, which serve as parameters of the system. To establish values for *K*′_2_ and *K*′_4_ from published transient calcium binding measurements [14], we paired the fastest measured on-rate with the two measured off rates. Thus, based on the ratio of measured rates (association/dissociation), 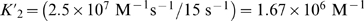 and 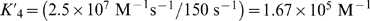 (Table 2). From the ratio, 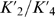 and conservation at equilibrium (Fig. 2), values for 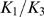 and 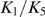 can be established (Table 2). This leaves *K*_1_ as the only adjustable parameter in the absence of myosin (Fig. 2).

**Table 2:** Summary of Parameters.

Parameter	Value	Comments
*K*_0_	1	Composite of constants for myosin-actin interaction and coupling to the position of Tm; adjustable parameter for modeling.
*K*_1_	500	Composite of constants for Tn-actin interaction (no bound calcium) and coupling to the position of Tm; constrained by 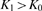 ; adjustable parameter for modeling.
*K*_3_	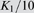	Composite of constants for Tn-actin interaction (one bound calcium) and coupling to the position of Tm; value given by 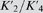
*K*_5_	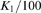	Composite of constants for Tn-actin interaction (two bound calcium) and coupling to the position of Tm; value given by ( 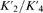 )^2^
*K*′_2_	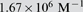	Constant (evaluated from [14])
*K*′_4_	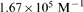	Constant (evaluated from [14])
*K*_2_	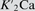	Independent variable; allows input of calcium (Ca) for computation
*K*_4_	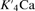	Independent variable; allows input of calcium (Ca) for computation
*α*	3–6	Maximum number of unsupported Tm subunits of a segment (evaluated from [22])
*n*	1–5	Number of coupled myosin per super segment; modeling results suggest 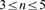 .
*p*	0–1	Adjustable parameter of the fraction of native Tn
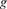	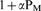	Number of Tm subunits in a segment
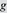 _max_	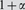	Maximum segment length
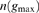	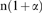	Maximum super segment length

The myosin-dependent parameters, *α* and *n*, are estimated from structural considerations. Favorable reconstructions of myosin-decorated thin filaments reveal 120–300 nm of unsupported Tm in Position M [22],which corresponds to 4–7 Tm subunits (based on 38.7 nm per Tm subunit). Thus, the ranges of *g_max_* and *α* are given by 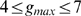 and 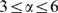 respectively (Table 2). The range of *n* is constrained by the fact that *n* segments of length *g_max_* cannot exceed the number of Tm units within the overlap region of a single thin filament (∼20). Hence, *n* cannot exceed 5 (Table 2).

**Figure 3:**
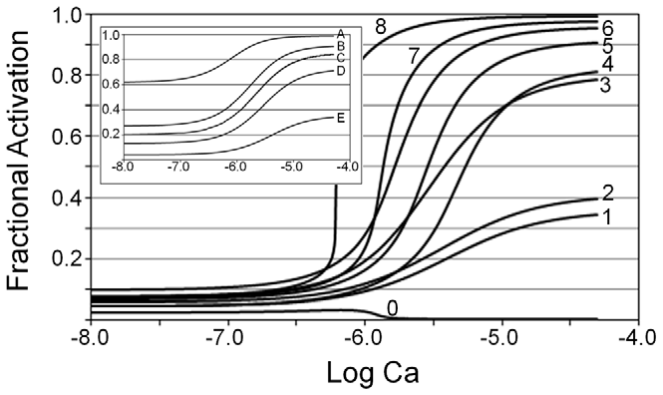
Factors that determine cooperative activation by calcium. Activation is calculated as the sum of the dependent variables C and M (Table 1) by solving Eqs. 9, 18, 21, and 22 given arbitrary calcium. Inset. Non-cooperative fractional activation in the absence of myosin. Myosin is excluded by setting the parameter *K*_0_ (Table 2) to zero. Fractional Activation is the dependent variable C (Table 1) as a function of calcium. Inset adjustable parameters: 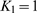 (Curve A); 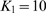 (Curve B); 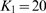 (Curve C); 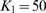 (Curve D); 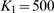 (Curve E). Outset. Myosin induces cooperative fractional activation. All curves except Curve 1 include myosin contribution by setting the parameter, *K*_0_, to one; for visual comparison to a non-cooperative activation, Curve 1 is reproduced (Curve E; inset). Curves 2, 3, 5, 7, and 8 illustrate the effects of parameters that control cooperativity: Curves 2 and 3 compare the effects of varying *α* and *n* given fixed 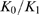 and Curves 5, 7, and 8 compare the effects of varying *n* given fixed *α* and 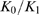. For constant *n* and *α* (Curves 4–6), increasing 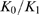 shifts the curves toward greater calcium sensitivity while the steepness remains nearly the same. Curve 0 shows the mole fraction of Tm in Position C as a function of calcium. Outset adjustable parameters: 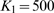, 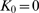 (Curve 1); 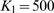, 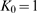, 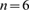, 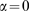 (Curve 2); 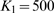, 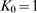, 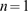, 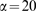 (Curve 3); 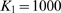, 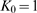, 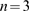, 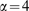 (Curve 4); 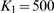, 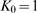, 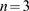, 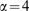 (Curve 5); 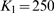, 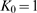, 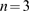, 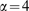 (Curve 6); 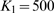, 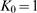, 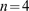, 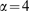 (Curve 7); 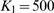, 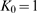, 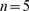, 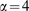 (Curve 8). Outset constants: 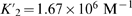, 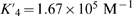.

We studied the behavior of the model restricted to Positions B and C in the absence of myosin. In the absence of myosin, activation is taken as the mole fraction of Tm in Position C. We solved for C using Eqs. Eqs. 9, 18, 21 and 22 given an arbitrary calcium. To prevent Position M, we set *K*_0_ to zero, which means *K*_1_ becomes the sole adjustable parameter of the simulation. Three characteristics of the curves vary with *K*_1_, namely, the baseline (activation at lowest calcium), the extent (difference between baseline and plateau), and sensitivity to calcium (calcium concentration at the midpoint between minimum and maximum). As *K*_1_ is increased, the fractional activation becomes less sensitive to calcium and the baseline decreases (Fig. 3, inset). The extent of activation has a maximum near Curve C (inset, Fig. 3), which is the simulation consistent with the results of particle tracking [19]. Biochemical results estimate the range of activation to be approximately 5% (baseline)–50% (plateau) [31], which is given by Curve E (inset, Fig. 3). In addition, resting activation of skeletal muscle fibers is estimated to be 6.5% and 10% from measurements at low calcium of residual ATPase and muscle stiffness (correcting for osmotic swelling) respectively [32], [33]. In summary, the simulations without myosin shows that calcium activation is not cooperative, calcium sensitivity is not uniquely dependent on calcium binding, the full range of calcium activation cannot be achieved in the absence of myosin given physiological constants for calcium binding, and the extent of calcium activation may be <50% to achieve a low baseline. For subsequent simulations, we uses the conditions consistent with biochemical and physiological measurements [31]–[33], which are those that generated a low baseline activation (Curve E).

To determine the criteria for a cooperative response, we test the two myosin-dependent parameters, *α* and *n* (Eq. 9), individually. Compared with a non-cooperative response (Curve 1; Fig. 3), a cooperative response is not observed for 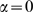 even when 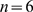 (Curve 2; Fig. 3), whereas a cooperative response is achieved with 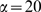 and 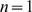 (Curve 3; Fig. 3); these results are consistent with *α* and not *n* being crucial for sigmoidal activation. A synergistic increase in steepness of the curves results from the combination of 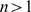 and 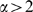 (Curves 4–8; Fig. 3). For a given 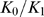 and *α*, increasing *n* generates a progressively steeper calcium activation, greater extent of calcium activation, and greater calcium sensitivity (Curves 5, 7, 8; Fig. 3). Increasing 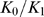, given constant *n* and *α*, cause the curves to shift toward greater calcium sensitivity and extent of activation, but the steepness does not change significantly (Curves 4–6; Fig. 3). Tm in Position C declines to zero as calcium is increased (Curve 0; Fig. 3). Thus, Position C contributes only to baseline activation and most of the transitions during calcium activation are between Positions B and M.

We investigated combinations of *n* and *α* that generate the greatest extent of activation. Approximately 90% of the full extent of activation (∼5% to ∼95%) is achieved not only with 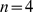 and 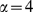 (Curve 7; Fig. 3), but also with 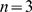 and 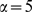 and 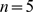 and 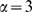 (not shown). The maximum super segment (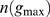; Table 2) computed from these pairs of parameters converges on a value of ∼20 Tm subunits. Further investigation shows that the curves become too steep to calculate using standard software packages when 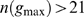, and the extent of activation becomes progressively reduced when 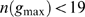. These results compare favorably with the region of thin filament overlap with thick filaments estimated to be ∼20 Tm subunits.

**Figure 4:**
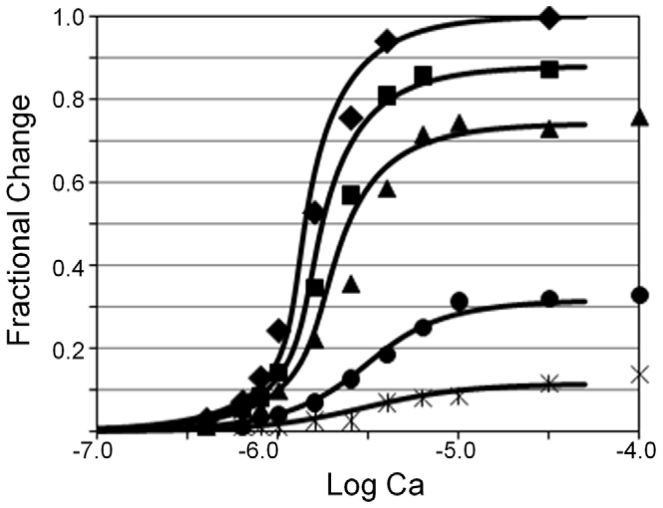
Fit of isometric tension data. Tension data are taken from [28]. The symbols represent the fractional change in isometric tension of skeletal muscle fibers reconstituted with a mixture of wild-type Tn and mutant Tn unable to bind calcium; the mole fraction of wild-type Tn is indicated as, filled diamond (100%), square (80%), trianlge (60%), circle (20%), cross (15%). Theoretical curves represent the mole fraction of Tm in Positions C and M, which is a measure of fractional activation. C and M are determined for arbitrary calcium by solving Eqs. 9, 18, 21, and 22. We normalized the raw simulations by subtracting the baseline (value at lowest calcium) and setting the maximum value (100% wild-type Tn at saturating calcium) equal to 1. The raw simulation with 100% wild-type Tn appears in Fig. 3 (Curve 7). Curves from left to right were generated with the following percentages of wild-type Tn: (left to right) 100% (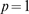), 83% (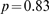), 70% (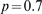), 33% (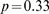), 15% (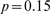). Adjustable parameters: *p*. Constants: 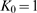, 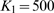, 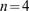, 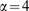, 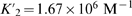, 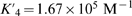.

Mutant Tn incapable of binding calcium is shown not only to reduce maximum tension, as expected, but to also reduce the steepness and calcium sensitivity of the curves generated by steady-state additions of calcium [28]. Given the simulations above, we asked whether all the effects of mutant Tn could be reproduced with our model. Fitting data from native muscle fibers requires combinations of *n* and *α* in which 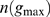 is 20 as noted above, owing to the steepness of the steady-state tension response to calcium (diamond, Fig. 4). To simulate mutant Tn that cannot bind calcium, we introduce a new coupled state of Tn, B^−^, which binds actin identically with native Tn devoid of calcium (*K*_1_; Eqs. 17 and 18; Table 1). Given the mole fraction of wild-type Tn (*p*; Table 2) as the only adjustable parameter, the model accurately simulates the calcium sensitivity and steepness of the data (Fig. 4). Although simulations are consistent with a reduction in maximum tension, the model overestimates the amount of wild-type Tn required to achieve maximum tension, by 5, 10, and 13% for reconstitutions with 80, 60, and 20% wild-type Tn, respectively (Fig. 4). However, in other simulations (not shown), all but the 20% data were fit to the accuracy of the measurement without overestimation when the actin binding constant of mutant Tn was reduced ∼50%. The affinity of mutant Tn for actin was not measured in the experimental study [28].

**Figure 5:**
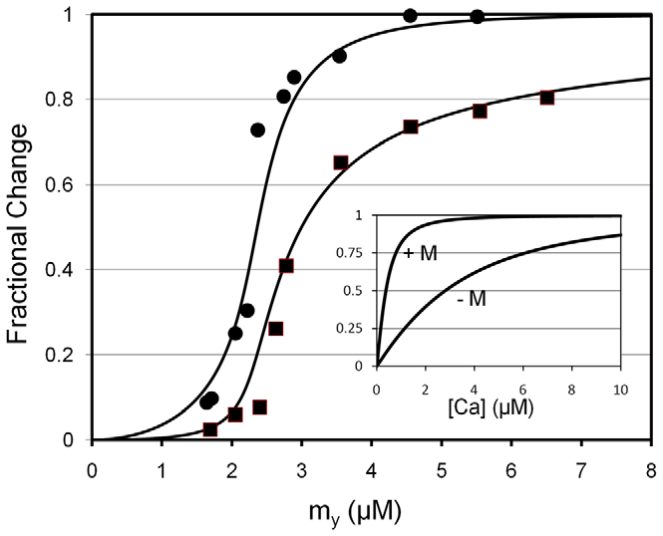
Relationship of IANBD fluorescence data and total myosin binding. All data are replotted from Trybus and Taylor [16]. Fluorescence data (circles) are fit by eye with Eq. 24, given 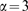 and 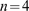; the curve through the data is generated by Eq. 23 using 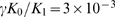. Total myosin binding data (squares) are fit with a curve representing the sum of coupled and free myosin binding using Eq. 25. As inputs to Eq. 25, coupled myosin binding is given by the change in fluorescence generated by Eq. 23 (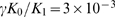) and the free myosin binding is generated by simple mass action (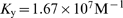 [4]; Eq. 26). Inset. Simulated calcium binding to Tn is non-cooperative. The sum of B_2_, B_3_, T_2_, and T_3_ (Table 1), which represents the total calcium bound to Tn, is plotted on the Y-axis. Values for these dependent variables were determined by solving Eqs. 9, 18, 21, 22 for arbitrary calcium. Total calcium binding with zero myosin (−M) and saturating myosin (+M) was simulated using *K*_0_ = 0 and 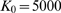, respectively. Fixed inset parameters: 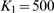, 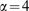, 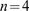, 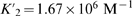, 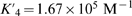.

To test for consistency with results of reconstitution experiments, we simulated myosin binding data [16]. Our working assumption is that the published fluorescence data [16] represents myosin coupling, because the movement of Tm to Position M accompanies the fluorescence change. Using Eq. 24, we obtain a best visual fit of the fluorescence data (circles, Fig. 5) when 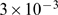 is the value of the dependent variable, 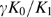. Evaluation of factor, γ, which accounts for variable bound myosin, is required for a valid comparison with the results that are obtained with myosin constrained by the muscle lattice. Nevertheless, it is encouraging that 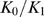 is typically 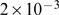 (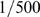) in the simulations of the intact muscle above (Figs. 3 and 4).

Total myosin binding in our model is the sum of two distinct populations of myosin, namely, coupled and non-coupled. Coupled myosin binds cooperatively and is given by the change in fluorescence (ΔF; Eq. 23). The binding of coupled myosin exposes the bulk of the actin binding sites for association with uncoupled myosin, which binds non-cooperatively (θ; Eq. 26). To simulate the behavior of the two populations of myosin, a total binding isotherm (Θ; Fig. 5) is generated with Eq. 25, given inputs by both ΔF and θ. Best fits of the experimental data are achieved with 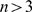 owing to the steepness of the response (data not shown), although data confidence is not sufficient to exclude any fits we obtained for 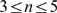.

Calcium binding to regulated actin has been shown to be non-cooperative both in the absence and presence of myosin [13], [14]. To test our model for cooperative calcium binding, we solved Eq. 9, 18, 21, and 22 for the sum of the calcium bound states B_2_, B_3_, T_2_, and T_3_ (Table 1), given an arbitrary free calcium. The binding in the absence of myosin was simulated by setting *K*_0_ to zero. For saturating myosin, we chose a value of *K*_0_ in preliminary simulations that was more than necessary to produce 100% activation for the full range of calcium (legend; inset; Fig. 5). The results of the simulations show that the predicted calcium binding curves are non-cooperative both in the presence and absence of myosin (inset; Fig. 5), as is expected [13], [14]. A shift of the simulated binding curves to higher affinity is found when myosin is included (inset; Fig. 5). The myosin-induced shift to higher affinity binding fits well with experimental observation [14].

## Discussion

We demonstrated that a model based on well-described biochemical reactions and the known positions of Tm can fit disparate data related to muscle regulation. The requirement for a super segment follows from our simulations of cooperative calcium activation and experimental observation. Although a sigmoidal dependence on calcium can be achieved for a single segment (Curve 3; Fig. 3), experiments show that a single myosin cannot move the entire Tm strand of a thin filament and, instead, demonstrate that the likely length of a single segment is represented by 3–7 Tm subunits [22]. To be consistent with this segment size and with the steepness of experimental activation curves, our simulations requires simultaneous coupling by 3–5 myosin. The length of a super segment is determined by the number of coupled myosin (separate segments) multiplied by the number of Tm subunits of a component segment. The best fit of experimental data is consistent with a super segment length equal to the overlap region of the thin filament (∼20 Tm subunits).

Our work builds on previous descriptions of coupling of binding energy to the work associated with the position of Tm [3], [26] and the uncoupling of myosin by the stiffening of Tm subunits [3], [7], [8], [26]. Previous models have fixed the length of the stiffened segment, which means that the free myosin is simply proportional to the coupled myosin. Free myosin has been previously modeled as a constant determined by the muscle lattice (c.f. Fig 5, [26]) and every Tm subunit in Position M has been modeled as supported by a coupled myosin [3], [26]. However, we propose a mechanism that enables free myosin to vary even as total myosin is fixed (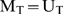; see Model Derivation above). By mass action, the free myosin stabilizes the rigid Tm structure and propagates an unsupported segment of multiple Tm subunits (Fig. 1). One advantage is that free myosin has unregulated access to actin binding sites for force production, whereas coupled myosin must expend energy on the position of Tm.

In the derivation, we assumed that *K*_B_ favors the actin-Tm interaction in Position C, but what if the equilibrium favors Position B. A recent reconstruction of reconstituted actin-Tm reveals that cardiac Tm occupies both Positions B and C when Tn is not associated and that association with Tn stabilizes Tm in either Positions B or C when in the absence or presence of calcium respectively [35]. These findings are consistent with a weak Tm-actin interaction that can be influenced by the interaction between Tn and Tm. Indeed, the fact that a fragment of Tn stabilizes Tm in Position B rather than Position C [36] suggests that Tm may acquire an interaction with actin in Position B, although this interaction would clearly be separate from the calcium-dependent Tn-actin interaction. Further study is required to fully resolve the question of Tm position absent the Tn-actin interaction, especially with regard to whether the movement of Tm between Positions B and C is passive or calcium dependent. The outcome of these studies will determine *K*_B_ in our model. Indeed, if it were shown that calcium binding promotes the transition to Position C, *K*_B_ would then become calcium dependent. Although this would complicate our model, especially with regard to filament sliding, the main conclusions of the present study would not change because Position C is shown here not to play a large role in the modeling of isometric tension (Curve 0; Fig. 3) and because the Tn-actin interaction dominates the stability of Tm in Position B.

Our simulations are the first to suggest that a balance between protein-protein interactions (expressed mathematically by 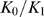; Table 2) determines calcium sensitivity even as the calcium binding parameters are held constant (Fig. 3). A bimolecular interaction between Tn and actin is the basis for this effect; this can be demonstrated by comparing our model with others that do not explicitly express the Tn-actin interaction as bimolecular [3], [4], [6]. In the absence of calcium, it can be shown that the mole fraction of Tm in Position C is a simple proportion, 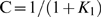, if a conformational change is assumed, but is a quadratic, 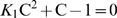, if the reaction is considered bimolecular. Similar relationships can be derived for infinite calcium and are presumed to occur for arbitrary calcium. A model based on a conformational change must alter calcium binding affinities to account for changes in calcium sensitivity. However, mutant Tn does not bind calcium, making it difficult to imagine how the mutant Tn can alter the calcium binding affinity of the wild-type Tn that is responsible for calcium-dependent tension generation [28]. Our simulations suggest that reduced calcium sensitivity results from an inability of mutant Tn to weaken its interaction with actin independently of calcium binding to wild-type Tn (Fig. 4). In addition, the simulated parallel shifts in calcium sensitivity when 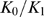 is altered (Fig. 3), may be useful in analyzing the effects of altered thin and thick filament interactions that occur as a result of changing physiologic and signal transduction conditions.

The super segment mechanism we propose is consistent with all present day actin-based contractile systems with or without Tn, including striated muscle, smooth muscle, and non-muscle, provided that Tm and filamentous myosin (myosin II) are involved. It is interesting to note that the calcium-dependent, steady-state tension relationship is sigmoidal for the molluscan adductor muscle [34], even though this muscle does not contain Tn and is regulated by calcium binding directly to myosin. We suggest that calcium binding to molluscan myosin regulates, P_M_, the probability that myosin can couple, and a similar mechanism holds for more typical contractile systems regulated by the phosphorylation of myosin. The evolution of the cooperative mechanism we propose would likely require that thick filaments coexist to deliver myosin to the locations of each Tm subunit simultaneously. Given an ancestral Tm gene, Tn could have evolved after the cooperative mechanism as a means of stabilizing the blocking position of Tm.

A tension derived from the myosin-induced displacement of Tm may provide a plausible mechanism for super segment formation. Muscle stretch has been shown to recruit myosin attachment to thin filaments and to move tropomyosin to Position M at sub-maximal calcium [37]. The energy of the external stretch could induce the rigid conformation of Tm, but myosin attachment would be required to stabilize Tm in Position M. In contrast to the stretch activation phase, which does not require cycling myosin, the steady-state tension phase subsequent to the stretch must have myosin capable of cycling [37], [38]. These observations are consistent in our model with the requirement for a steady-state supply of free myosin (cycling myosin) to sustain the coupled state. Multiple myosin working in concert on the position of Tm could impose an axial stretch on Tm, resulting in a reciprocal tension placed on myosin heads as they couple, thereby stabilizing the complex of Tm, myosin, and actin. A stretch-based mechanism of super segment formation is consistent with the large compliance observed for the thin filament [39], [40] and the proposal for a single mechanism to account for both calcium and stretch activation [41].

We suggest the following as potential concerns for the validity of our model. First, the length of Tm that overhangs myosin-decorated actin is the main evidence for a conformational change that stiffens Tm [22]. Further refinements of existing images or additional structural reconstitutions may reveal hidden myosin, as has been suggested [7]. Second, unless present particle-tracking measurements underestimate the mole fraction of Tm in Position B, our model would have to be modified. We can account for either the particle tracking data (Curve C; inset, Fig 3) [19] or biochemical measurements of activation (Curve E; inset, Fig. 3) [16], [31]. To account for both, the characteristics of Position C would have to be changed, but not necessarily the mechanisms describing the transitions between Positions B and C and Positions C and M. It should be noted that Tm in Position C does not play a large role in simulations of isometric conditions (Curve 0, Fig. 3). Third, the rate of Tm movement would have to exceed 1000 s^−1^ to not limit the expected rates of myosin recruitment and transitions between Positions C and M. We are encouraged that present measurements do support rapid transitions of Tm in response to myosin binding [42], but additional study is required to establish upper limits of rate. Additionally, a stiffened conformation of Tm would have to relax fast enough to not limit the decay of Position M. Fourth, a grossly inhomogeneous myosin distribution along the thin filament is the greatest concern for our model of isometric contraction, but variance in bound myosin is also a concern. Reconstructions of insect flight muscle demonstrate a periodicity of bound myosin that corresponds to the central location of each Tm subunit [11]. Statistical variation about 1.3 myosin heads per Tm subunit is low for the planar packing of thin and thick filaments of insect muscle [11] and may even be less for vertebrate muscle, which has trigonal symmetry. Fifth, the model presented here would be inconsistent with activation greater than that achievable by actin and Tm alone. Sixth, for our model to be valid, the affinity and kinetics of calcium binding to Tn in Positions C and M must be the same as those exhibited by pure Tn. To avoid ambiguity in assigning rate constants measured from two calcium binding sites [14], Tn with only one regulatory site would be required. Finally, we suggest that as future experiments become available, it should be possible to infer forward and reverse rates for all of our model's equilibrium constants.
